# Noncoding-RNA mediated high expression of zinc finger protein 268 suppresses clear cell renal cell carcinoma progression by promoting apoptosis and regulating immune cell infiltration

**DOI:** 10.1080/21655979.2022.2060787

**Published:** 2022-04-23

**Authors:** Keyi Wang, Yongzhe Gu, Jinliang Ni, Houliang Zhang, Yidi Wang, Yifan Zhang, Xianchao Sun, Tianyuan Xu, Weipu Mao, Bo Peng

**Affiliations:** a Department of Urology, Shanghai Tenth People’s Hospital, School of Medicine, Tongji University, Shanghai, Zha Bei Qu, China; b Department of Neurology, Shanghai Tenth People’s Hospital, School of Medicine, Tongji University, Shanghai, Zha Bei Qu, China; c Shanghai Clinical College, Anhui Medical University, Hefei, Anhui Province, China; d Department of Urology, Affiliated Zhongda Hospital of Southeast University, Nanjing, Jiangsu, China

**Keywords:** ZNF268, kidney renal clear cell carcinoma, prognosis, immune infiltration, noncoding RNA

## Abstract

Clear cell renal cell carcinoma (ccRCC) is one of the most common malignant kidney tumors with a poor prognosis. Accumulating evidence proves that zinc finger protein 268 (ZNF268) is associated with tumor progression, but the detailed regulatory functions of ZNF268 in ccRCC require further exploration. Thus, here we aim to characterize the role of ZNF268 in ccRCC. The clinical significance of ZNF268 was evaluated using The Cancer Genome Atlas (TCGA) and the Genotype-Tissue Expression (GTEx) databases. Subsequently, tumor-infiltrating immune cells, as well as upstream noncoding RNAs (ncRNAs) related to the tumor-suppressing function of ZNF268, were identified by *in silico* analyses. The expression of ZNF268 was significantly decreased in ccRCC samples compared with adjacent normal tissues. In addition, ZNF268 expression was negatively correlated with tumor progression and positively correlated with overall and disease-specific survival. TCGA and GTEx databases proved the potential tumor-suppressing function, which was measured both *in vitro* and *in vivo* after ZNF268 over-expression. Overexpression of ZNF268 effectively inhibited the proliferation, migration, invasion and promotied apoptosis of the Caki-1. The level of ZNF268 was positively related to the immune cell infiltration in the tumor. Moreover, we determined that the AC093157.1/miR-27a-3p axis can potentially regulate ZNF268 function in ccRCC. Our work describes a novel ncRNA-mediated ZNF268 function in ccRCC. ZNF268 acts as a tumor suppressor, and it is associated with apoptosis and immune cell infiltration in ccRCC.

## Graphical abstract

**Figure:**
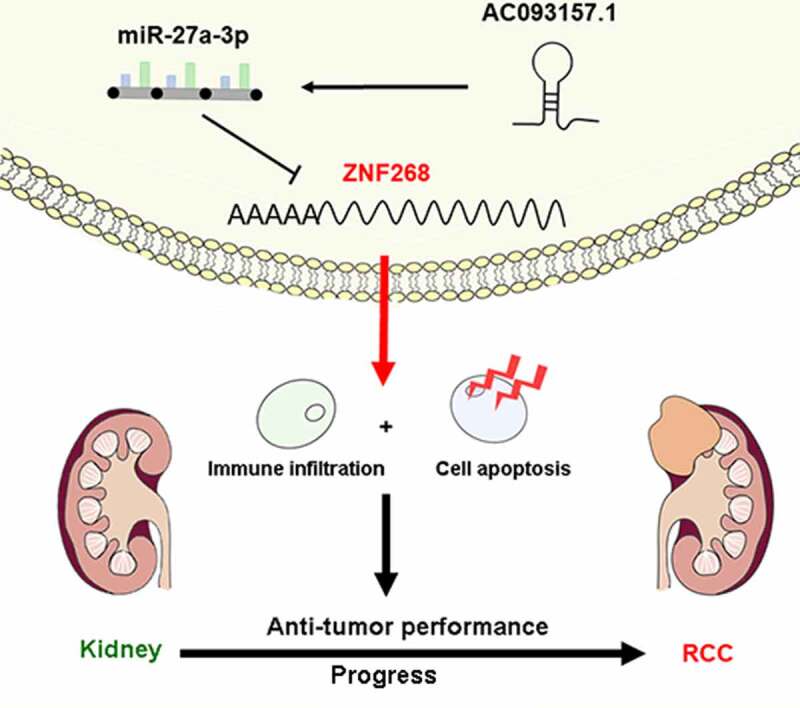

## Introduction

Clear cell renal cell carcinoma (ccRCC), one of the common malignant tumors in the genitourinary system, accounts for about 3% of all systemic malignant tumors[1]. It has been estimated that there will be 76,080 new cases of ccRCC and 13,780 deaths in the United States in 2021, rendering it the sixth most prevalent malignancy among men[2]. ccRCC mainly originates from epithelial cells in the renal tubules and accounts for approximately 90% of renal malignancies[3]. Currently, radical or partial nephrectomy is the most common treatment for early-stage ccRCC. Nevertheless, ccRCC has a high propensity for recurrence, with metastatic lesions found in 50% of patients at postoperative follow-up[4]. Furthermore, ccRCC becomes resistant to chemotherapy and radiotherapy once the recurrence or metastasis happens in patients[5]. Therefore, it is imperative to explore new effective therapeutic targets and reliable prognostic biomarkers for ccRCC.

The development of ccRCC involves multiple genetic mutations and dysregulation of epigenetic pathways[6]. The Krüppel-associated box (KRAB)-containing zinc finger proteins are the largest family of transcriptional repressors in mammals[7]. This family is characterized by an N-terminal KRAB domain and a C-terminal array of two to 40 C2H2 zinc fingers. Zinc finger protein 268 (ZNF268) is a common KRAB-containing zinc finger protein that presents two different protein isoforms: ZNF268a and ZNF268b2[8]. Previous studies have shown that ZNF268 is also associated with human fetal liver development and hematologic malignancies [9,10].

T lymphocytes occupy an irreplaceable position in the human immune system, and they can influence the development of many diseases, such as autoimmunity, by regulating the body’s immune responses[11]. Recent studies suggested that preoperative levels of CD3^+^ T cells and peripheral blood CD4^+^/CD8^+^ ratios are independent predictors of prognosis in patients with renal cancer[12]. Moreover, high infiltration of CXCL13^+^ CD8^+^ T cells leads to impaired immunity of CD8^+^ cells and immune escape of tumor cells. Immune escape promotes tumor proliferation and is associated with a poor prognosis of ccRCC[13].

Nevertheless, the relationship regarding the relevance of ZNF268 expression, prognosis, and tumor immune infiltration in ccRCC remains undetermined. In this study, we analyzed the expression and function of ZNF268 in ccRCC and verified the regulatory effect of ZNF268 on tumor progression both *in vitro* and *in vivo*. In addition, we explored the association between ZNF268 expression and immune cell infiltration in ccRCC with a special focus on central memory T (T_CM_) cells and T helper (Th) cells. Finally, we determined the AC093157.1/miR-27a-3p axis as the potential noncoding RNA (ncRNA)-mediated upstream pathway of ZNF268 in ccRCC. In summary, this work revealed that AC093157.1/miR-27a-3p/ZNF268 axis promoting cell apoptosis and immune cell tumor infiltration for tumor inhibition in ccRCC.

## methods and materials

### RNA-sequencing data and bioinformatics analysis

The mRNA expression data and clinical information were retrieved from The Cancer Genome Atlas (TCGA) database (https://genome-cancer.ucsc.edu/). This dataset included 539 cases of RCC patients and 72 matched adjacent tissues. Additional 28 normal cases were obtained from The Genotype-Tissue Expression (GTEx) database from UCSC Xena (https://xenabrowser.net/datapages/). The data from TCGA database were transferred from fragments per kilobase per million (FPKM) into transcripts per million (TPM) format for further expression analysis; the data downloaded from GTEx database was TPM format and directly included in the subsequent analysis. The normalized data from the two databases were analyzed by R software using limma package (http://www.r-project.org)[14]. A *P*-value < 0.05 was set as statistically significant.

### Cell culture and transfection

The human cell line Caki-1 from the Cell Bank of the Chinese Academy of Sciences (Shanghai, China) was chosen for *in vitro* studies due to its low ZNF268 expression. Caki-1 cells were cultured in RMPI 1640 (Gibco, USA) supplemented with 10% Fetal Bovine Serum (FBS, Hyclone, USA) and 1% Penicillin/Streptomycin (P/S, YEASEN, China) at 37°C in 5% CO_2_. Lentiviral oe-ZNF268 with relative nonspecific negative control (NC) was purchased from GENECHEM Incorporation (China); the miR-27a-3p inhibitor and the small interfering targeting AC093157.1 (si-AC09315.1) with corresponding NC were obtained from RiboBio (Guangzhou, China). All the reagents were transfected in the cells using jetPRIME (YEASEN, China) according to the manufacturer’s protocol. Detailed sequences of si-AC09315.1 are listed in the ***Table S1***.

### RNA extraction and quantitative real-time PCR

Total RNA was isolated using the Trizol reagent (Invitrogen, USA) according to the manufacturer’s protocol. RNA concentration was measured using a Nanodrop 2000 spectrophotometer (Thermo Scientific, USA). After cDNA synthesis, the relative expression of ZNF268, miR-27a-3p and AC093157.1 was measured by RT-qPCR using primers specific for ZNF268 (Sangon Biotech, Shanghai, China), miR-27a-3p (RiboBio, Guangzhou, China) and AC093157.1 (Sangon Biotech, Shanghai, China) according to the instructions. GAPDH was used as a control to correct for differences in template input, and the relative expression was calculated by the 2^−ΔCT^ method. Detailed primer sequences are listed in the ***Table S2.***

### Western blot

Western blot experiments were performed as previously reported[15]. Cells were lysed with RIPA buffer (YEASEN, China) containing PMSF (Beyotime, China). After determining the protein concentration using the BCA protein kit (Thermo Scientific, USA), the protein lysate was mixed with 5 X SDS loading buffer (EpiZyme, China) and heated at 100°C for 10 min. A total of 30 μg of proteins were separated and transferred to a PVDF membrane. Then, the membrane blocked by 5% nonfat milk for 1 h was incubated with an anti-ZNF268 antibody (1:1000; Abmart, China), anti-GAPDH antibody (1:1000; Abcam, USA), anti-BCL-2 antibody (1:1000; Abcam, USA) and anti-Bax antibody (1:1000; Abcam, USA) in 4°C overnight. And the membrane was dyed by ECL after the co-incubation of the secondary antibody (1:5000; Jackson immunoresearch, USA) for 1 h.

### EdU analysis for cell proliferation

Cells were cultured in 48-well plates after the transfection with oe-ZNF268 or miR-27a-3p-inhibitor for 24 h and then treated with 10 μM EdU (Beyotime, China) for 2 h at 37°C in 5% CO_2_. Pre-treated cells were fixed with 4% paraformaldehyde for the next penetration stage, assisted by PBS buffer containing Triton X-100 (0.5%). After washing with PBS/0.3% BSA, the cells were incubated with Alexa Fluor 488 and DAPI in the dark. EdU results were visualized using a Leica DM6 B upright microscope system (Leica, Germany).

### Colony formation, wound healing, and transwell assay

A colony formation assay was conducted to measure cell proliferation. A density of 5^10^2^ cells was plated in a 6-well plate and stained with crystal violet after 2 weeks. Wound healing experiments were performed to assess the migration ability of the cells after transfection. Wound width was measured at times 0 h and 24 h after the scrape. For the transwell assay, 5^10^4^ cells in 200 μL serum-free medium were cultured in the upper chambers with (invasion) or without (migration) matrigel, and 500 μL medium with 10% FBS was added to the lower chambers. The upper surface was gently rubbed with a cotton swab 24 h after incubation, and the lower surface was stained and imaged.

### Cell apoptosis assay

Cell apoptosis was examined by flow cytometry with the Annexin V-FITC Apoptosis Kit (BD Biosciences, Erembodegem, Belgium). All the procedure were conducted consisting with the manufacturer’s instructions. Briefly, the pretreated cells were washed by PBS twice to obtain the sedimentation. Then, the cells were stained by fluorescein isothiocyanate (FITC) and propidium iodide (PI) solution for 15 min at room temperature. Finally, the apoptosis rate was measured by the BD FACS Calibur (Beckman Coulter, CA, USA).

### Xenograft tumor model

The xenograft tumor experiment was approved by the Animal Ethics Committee of Shanghai Tenth People’s Hospital (SHDSYY-2020-1726). A total of 3^10^6^ cells (OE-ZNF268 and NC separately) washed twice by phosphate buffer saline (PBS) and diluted in saline (100 μl) were injected subcutaneously into the flanks of NOD-SCID mice (3 ~ 5 weeks; Shanghai Model Organisms Center, Inc, Chins) to assess tumor growth with six mice per group. Tumor volume was monitored every three days, and mice were sacrificed three weeks later. The method for calculating tumor volume complied with the following equation: Volume = length * width[2] * 0.5. The animal study was conducted in accordance with the guidelines from the Animal Care and Use Committee of Shanghai Tenth Peoples’ Hospital.

### Immunohistochemical (IHC) staining

Fresh tumor tissues were obtained from the euthanized mice after three weeks and foxed in 4% paraformaldehyde. After dehydration of ethanol, the tissues were embedded in paraffin. The tissues were sliced into 4 μm slides and immunohistochemically stained following a previously reported protocol[15]. The slides were incubated with anti-ZNF268 antibody for ZNF268 measurements. The images were photographed using the microscope (Leica Microsystems, Mannheim, Germany).

### Immune infiltration analysis

The immune infiltration analysis was performed as previously reported[16]. Twenty-four types of immune cells were included in the analysis. And the single sample gene set enrichment analysis (ssGSEA) were conducted with the R package GSVA (version 3.6) (http://www.bioconductor.org/packages/release/bioc/html/GSVA.html).The relationship between ZNF268 expression and immune cell infiltration was determined by the Spearman and Wilcoxon rank-sum test.

### Candidate ncRNA prediction

Carious target gene predicting databases (miRWalk, microRNA, TargetScan, and StarBase) were used to investigate potential upstream binding miRNAs. A miRNA was considered a binding candidate upon agreement between the four programs. As for lncRNA, we evaluated the association with miRNA through StarBase and LncBase. Furthermore, the related analysis was performed as the above methods.

### Luciferase reporter assay

To confirm ZNF268 was a direct target of miR-27a-3p, luciferase reporter assay was conducted in our study. The constructs containing wild-type or mutant ZNF268-miR-27a-3p were cloned into luciferase gene through pmirGLO vectors (Promega Corporation, Madison, WI, USA). These vectors were transfected to the 293 T cells by Lipofectamine 2000 (Invitrogen; Thermo Fisher Scientific, Inc.) for 24 h. Then, the firefly and Renilla luciferase intensity was measured using the dual luciferase reporter assay system (Promega, Massachusetts, USA). And the firefly to Renilla luciferase ratios were calculated per well and repeated three times.

## Statistical analysis

All analyses were performed using R (v.3.6.3) and GraphPad Prism software (Version 6.0, GraphPad Prism Software Inc., San Diego, CA). Chi-square tests, Fisher exact tests, and logistic regression were conducted to investigate the relationship between clinical features and ZNF268, miR-27a-3p and AC093157.1 as previously reported[16]. Kaplan-Meier analysis was used for the evaluation of TCGA patient survival rates. The results were considered statistically significant with a *P*-value < 0.05.

## Results

The ZNF268 anti-tumor function was investigated in this study for the potential clinical application of ccRCC therapy. Based on the experiments both *in vitro* and *in vivo*, it was exhibited that ZNF268 overexpression effectively suppressed proliferation, migration, and invasion of Caki-1 cells, and inhibited tumor progression. It was believed that the increasing apoptosis and immune cell infiltration contributed to the anti-tumor performances which proved by the results of *in vitro* and *in silico* analyses. Additionally, the upstream ncRNA were further investigated through various databases and the AC093157.1/miR-27a-3p axis was considered as the potential ZNF268 regulating mechanism with the confirmation of experimental results. This study revealed ncRNA mediated ZNF268 functioned well as the tumor suppressor by promoting cell apoptosis and immune cell tumor infiltration in ccRCC.

### ZNF268 expression is related to poor clinicopathological features of ccRCC

**Figure 1.**
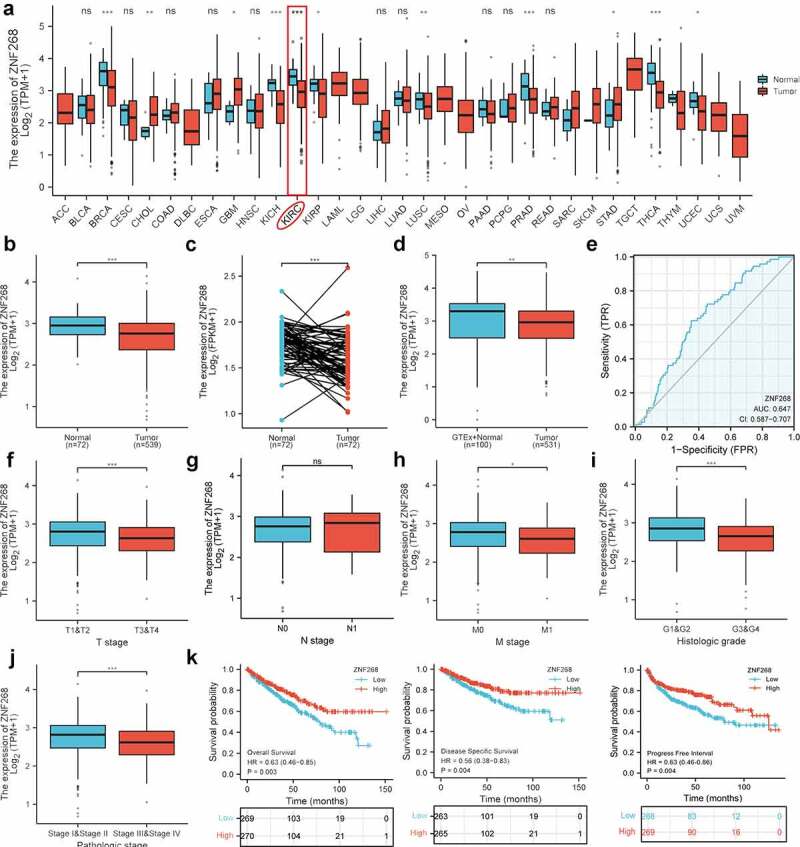
**Expression analysis and clinicopathological characteristics of ZNF268 in clear cell renal cell carcinoma (ccRCC)**. (a) Expression of ZNF268 in 33 kinds of human cancers based on the TCGA database; among the renal cancer, the KICH referred as kidney chromophobe, KIRC as kidney renal clear cell carcinoma and KIRP as kidney renal papillary cell carcinoma. Expression of ZNF268 in ccRCC tissues and adjacent normal tissues (b), and the matched analysis (c). Expression of ZNF268 in normal tissues of GTEx combined with TCGA and ccRCC tissues (d) and the ROC curve of ZNF268 distinguishing ccRCC tissues (e). (f-j) Relationship between ZNF268 expression and clinicopathological features in ccRCC, including TNM-stage, tumor grade and stage. (k) Kaplan-Meier curves for overall survival, disease-specific survival and progress free interval. (Data represent means ± SD, ns represents no statistical difference, *p < 0.05, **p < 0.01 and ***p < 0.001).

First, we investigated the expression of ZNF268 in 33 types of human cancer (Figure 1a). The results proved that ZNF268 was significantly downregulated in ccRCC samples, indicating that the low ZNF268 expression might be related to carcinogenesis. The expression data from 539 ccRCC patients and 72 adjacent tissues was further measured. These results corroborated that ZNF268 was highly expressed in normal tissues (*P* < 0.001; Figure 1b), which was also confirmed by the matched analysis (*P* < 0.001; Figure 1c). In good agreement with this, the expression results from GTEx combined TCGA database demonstrated that ZNF268 downregulation was associated with carcinogenesis (*P* < 0.001; Figure 1d). The receiver operating characteristic (ROC) curve was conducted to determine the effectiveness of ZNF268 expression in distinguishing ccRCC tissues from normal tissues based on the previous report (Figure 1e)[17]. Additionally, the relationships between ZNF268 expression and VEGFA/VHL, the biomarkers of ccRCC, were also investigated (***Figure S1A, B***). It was shown that ZNF268 expression was positively correlated with VEGFA/VHL expression, further suggesting the prediction value of ZNF268 in distinguishing ccRCC. Various detailed clinical features of the 539 ccRCC patients are listed in Table 1. There are 269 low-expression patients and 279 high-expression patients based on the mean value of ZNF268 expression. Moreover, the relationship between ZNF268 expression with T-stage (*P* < 0.001), N-stage (*P* > 0.050), M-stage (*P* = 0.080), pathologic stage (*P* < 0.001) and histologic grade (*P* < 0.001) was measured with the results indicating that low ZNF268 expression was accompanied with advanced tumor clinicopathological features (figure 1f-j and Table 1). Kaplan-Meier analysis results also proved that ZNF268 expression was positively correlated with overall survival (OS; *P* = 0.003), disease-specific survival (DSS; *P* = 0.004) and progress free interval (PFI; *P* = 0.004) (Figure 1k and Table 1), further proving the effective predictive performances of ZNF268.

**Table 1.** Clinical characteristics of KIRC patients

Characteristic	Low expression of ZNF268	High expression of ZNF268	*P*-value
n	269	270	
T stage, n (%)			< 0.001
T1	115 (21.3%)	163 (30.2%)	
T2	40 (7.4%)	31 (5.8%)	
T3	110 (20.4%)	69 (12.8%)	
T4	4 (0.7%)	7 (1.3%)	
N stage, n (%)			0.834
N0	120 (46.7%)	121 (47.1%)	
N1	7 (2.7%)	9 (3.5%)	
M stage, n (%)			0.080
M0	203 (40.1%)	225 (44.5%)	
M1	46 (9.1%)	32 (6.3%)	
Pathologic stage, n (%)			< 0.001
Stage I	112 (20.9%)	160 (29.9%)	
Stage II	32 (6%)	27 (5%)	
Stage III	75 (14%)	48 (9%)	
Stage IV	48 (9%)	34 (6.3%)	
Histologic grade, n (%)			< 0.001
G1	4 (0.8%)	10 (1.9%)	
G2	94 (17.7%)	141 (26.6%)	
G3	114 (21.5%)	93 (17.5%)	
G4	52 (9.8%)	23 (4.3%)	
OS event, n (%)			< 0.001
Alive	163 (30.2%)	203 (37.7%)	
Dead	106 (19.7%)	67 (12.4%)	
DSS event, n (%)			0.002
Alive	194 (36.7%)	226 (42.8%)	
Dead	69 (13.1%)	39 (7.4%)

### ZNF268 inhibits proliferation, migration, and invasion of Caki-1

**Figure 2.**
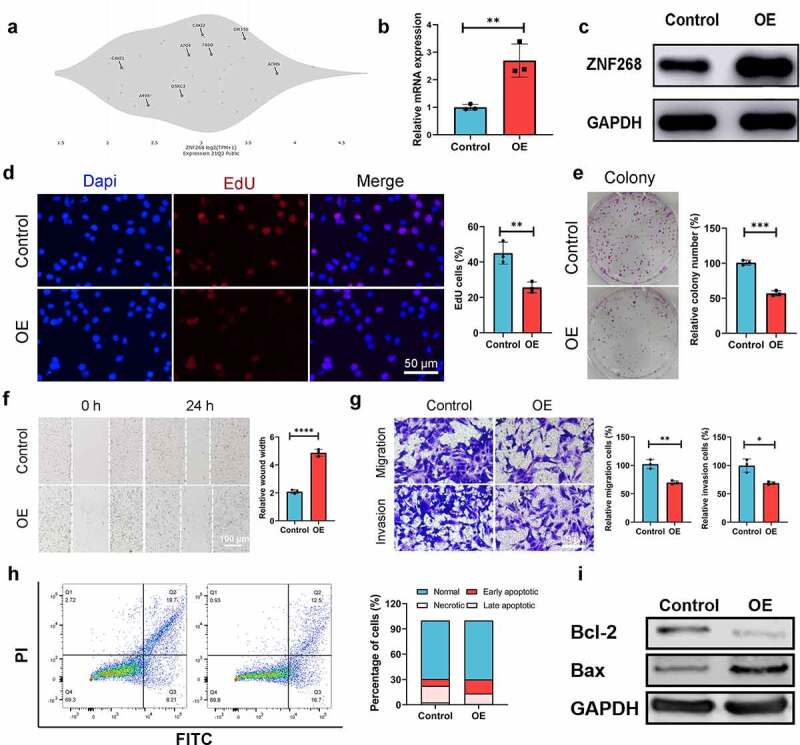
**Overexpression of ZNF268 inhibits cell proliferation, migration, and invasion by promoting apoptosis *in vitro***. (a) The expression differences of ZNF268 in ccRCC cell lines which exhibiting Caki-1 contained the higher expression. q-PCR (b) and Western blot (c) analysis of ZNF268 expression after transfection indicating the overexpression of ZNF268. (d) EdU results of ZNF268 overexpression in cell proliferation with the quantitative analysis in the right. (e) Results of the colony assay after ZNF268 overexpression in Caki-1 with the quantitative analysis in the right. (f) Wound-healing assay results after oe-ZNF268 transfection for migration measurements and the quantitative analysis in the right. (g) Transwell assay results for cell migration and invasion after oe-ZNF268 transfection with the quantitative analysis in the right. (h) Cell apoptosis results of Caki-1 after oe-ZNF268 transfection as measured through flow cytometry with the quantitative analysis in the right. (i) Western blot analysis of Bcl-2 and Bax expression in the two subgroups. (n = 3, data represent means ± SD, *p < 0.05, **p < 0.01, ***p < 0.001 and ****p< 0.0001).

The cell line of Caki-1 was selected for *in vitro* assays due to its low ZNF268 expression (Figure 2a; data downloaded from Depmap: https://depmap.org/portal/). To study the effects of ZNF268 on proliferation, migration, and invasion, we set up a ZNF268 overexpression system. Caki-1 cells were transfected with oe-ZNF268, and ZNF268 overexpression after transfection was validated by real-time PCR (mRNA) and Western blot (protein) (Figure 2b, c). Using this system, we observed that overexpression of ZNF268 reduced cell proliferation by EdU (Figure 2c), which was further proved by the results of colony experiments (Figure 2e). In addition, wound-healing and transwell assays demonstrated that ZNF268 upregulation contributed to the inhibition of cell migration and invasion *in vitro* (Figure 2d, f). Subsequently, this study measured the cell apoptosis by flow cytometry with the results showing that OE-ZNF268 increased the apoptosis of Cali-1 compared with NC group (Figure 2h). Meanwhile, the WB results of Bcl-2 and Bax expression proved the conclusion that OE-ZNF268 enhanced the cell apoptosis, which contributing to the anti-tumor performances (Figure 2i). In summary, the above results indicate that ZNF268 can function as a suppressor of cell proliferation, migration, and invasion in ccRCC.

### ZNF268 suppresses the Caki-1 growth through regulating immune infiltration

**Figure 3.**
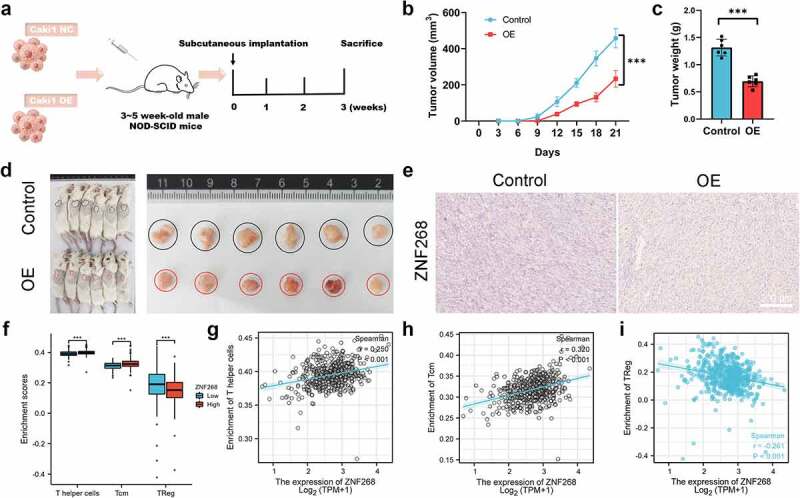
**ZNF268 suppresses Caki-1 proliferation *in vivo* through regulating immune cell infiltration** (a) Schematic representation of the Xenograft tumor model establishment. Analysis of tumor volume (b) and weight of xenograft tumors (c) (n = 6 each group). (d) Representative images of mice and the tumors with the two cells injection, and the tumors in the two subgroups. (e) Representative IHC staining images of ZNF268 in the two groups. (f) The relationship between ZNF268 expression and T helper cell, T_CM_ and Treg cells infiltration in ccRCC. ZNF268 expression is positively correlated with T helper (g) and T_CM_ (h) cells infiltration, which is contrary with Treg (i) cell infiltration. (Data represent means ± SD, ns represents no statistical difference, ***p < 0.001 and ****p < 0.0001).

The results from *in vitro* experiments revealed a potential tumor-suppressing function of ZNF268. We next explored ZNF268 function *in vivo* using a xenograft tumor model (Figure 3a). Caki-1/oe-ZNF268 cells were injected in the flank of NOD-SCID mice, and tumor volume was recorded every three days with the lower proliferation curve of OE-ZNF268 indicating the effective anti-tumor performances (Figure 3b). The results from tumor weight evidenced that overexpression of ZNF268 inhibited tumor growth *in vivo* (Figure 3c). Simultaneous, the representative images of xenograft tumors among the two subgroups also proved that ZNF268 functioned as a tumor suppressor in ccRCC (Figure 3d). Additionally, the IHC results of tumors proved the upregulation of ZNF268 *in vivo*, providing evidence of a potential tumor-suppressing effect (Figure 3e). To explore the potential mechanism of anti-tumor function *in vivo*, we further analyzed the relationships between ZNF268 expression and immune cell infiltration in ccRCC (***Figure S1C***). In the subgroup analysis, the lower ZNF268 expression group accompanied with the lower T helper cell and central memory (T_CM_) cell infiltration, while higher regulatory (T_reg_) cell infiltration (*P* < 0.001; Figure 3f). In addition, the results showed that ZNF268 expression was positively correlated with T_CM_ and T helper cell infiltration (*P* < 0.001; Figure 3g, h). On the contrary, ZNF268 expression was negatively correlated to the infiltration level of Treg cells (*P* < 0.001; Figure 3i) and NK CD56 bright cells (***Figure S1C***) The above results provided evidence that ZNF268 can activate the immune response in ccRCC to inhibit tumor growth.

### Prediction and analysis of upstream ncRNAs

**Figure 4.**
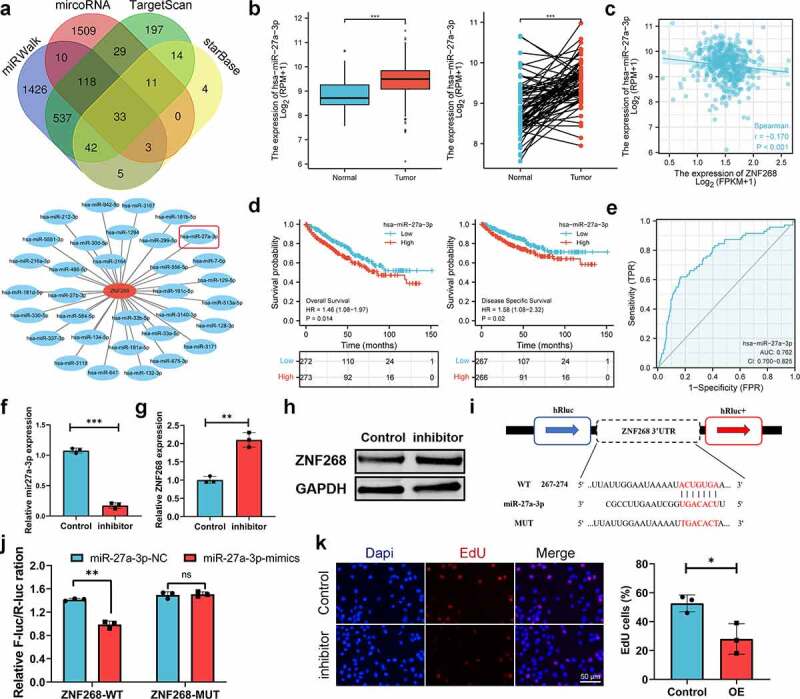
**MiR-27a-3p is selected as the upstream regulatory ncRNAs**. (a) miRNA selection based on four predicting programs is exhibited by the Venn diagram, and the miRNA-ZNF268 regulatory network is established by Cytoscape software. (b) Expression of miR-27a-3p in ccRCC tissues and adjacent normal tissues with the matched analysis on the right. (c) ZNF268 expression is negatively related to miR-27a-3p expression in ccRCC. Kaplan-Meier curves of miR-27a-3p for overall and disease-specific survival (d) with ROC curve distinguishing ccRCC tissues in the right (e). (f) q-PCR analysis of miR-27a-3p expression after miR-27a-3p-inhibitor transfection. q-PCR (g) and Western blot (h) analysis of ZNF268 expression after transfection indicating the miR-27a-3p regulating function of ZNF268. (i) EdU results of ZNF268 overexpression in cell proliferation with the quantitative analysis in the reight. (Data represent means ± SD, **p < 0.01 and ***p < 0.001).

**Figure 5.**
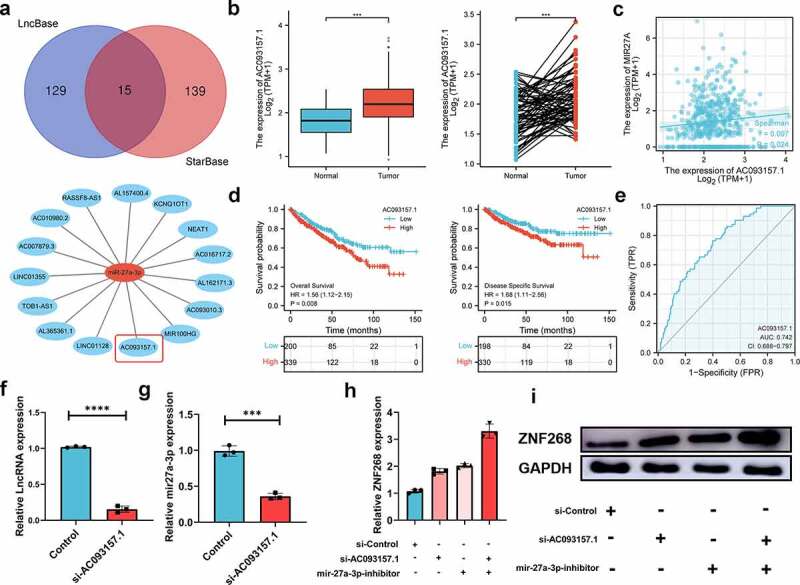
**LncRNA-AC093157.1 potential regulated the expression of miR-27a-3p**. (a) LncRNA selection based on two databases represented by a Venn diagram, and the LncRNA-miR-27a-3p regulatory network is established by Cytoscape software. (b) Expression of AC093157.1 in ccRCC tissues and adjacent normal tissues with the matched analysis in the right. (c) MiR-27a-3p overexpression is positively related to AC093157.1 expression in ccRCC. Kaplan-Meier curves of AC093157.1 for overall and disease-specific survival (d) with ROC curve distinguishing ccRCC tissues on the right (e). The expressions of AC093157.1 (f) and miR-27a-3p (g) in the two subgroups after si-AC093157.1 transfection. q-PCR (h) and Western blot (i) analysis of ZNF268 expression in the subgroups after transfection indicating the ZNF268 regulating function. (Data represent means ± SD, ***p < 0.001 and ****p < 0.0001).

With the broad recognition of ncRNAs as gene expression regulators, we aimed to predict the binding of potential upstream ncRNAs involved in ZNF268 regulation, including miRNA and lncRNA. miRNAs were first determined through four databases, with 33 candidates screened out (Figure 4a). A miRNA-ZNF268 regulatory network was established using Cytoscape for better visualization (Figure 4a). The negative correlation between miRNA and ZNF268 was considered the selection criteria for miRNA prediction (***Table S3***). And there were miR-27a-3p, miR-33a-5p and miR-33b-5p screened out (Figure 4c ***and S2A, B***). Next, the relationship between miRNA expressions and ccRCC patients’ OS were further measured to distinguish the potential upstream miRNA with miR-33a-5p and miR-33b-5p excluded in this work (***Figure S2C, D***). The investigations of miR-27a-3p expression in ccRCC were further conducted and showed the contrary expression with ZNF268 in both total and matched analysis (all *P* < 0.001; Figure 4b). The results from ***Figure S3A-D*** demonstrated that miR-27a-3p expression was related with T-stage (*P* < 0.01), N-stage (*P* < 0.05), M-stage (*P* < 0.001) and pathological stage (*P* < 0.001), indicating that high miR-27a-3p expression might be associated with advanced tumor clinicopathological features. Kaplan-Meier analysis results also showed that miR-27a-3p expression was negatively correlated with OS, DSS and PFI (Figure 4d ***and S3E***). The ROC curve was used to determine the effectiveness of miR-27a-3p expression in distinguishing ccRCC tissues from normal tissues with AUC as 0.762, indicating the effective predicting function of miR-27a-3p (Figure 4d). Meanwhile, the function of miR-27a-3p in ccRCC was explored by the miR-27a-3p inhibitor. The results of q-PCR indicated that miR-27a-3p expression were effectively inhibited by the miR-27a-3p inhibitor (Figure 4f). And the expression of ZNF268 was correspondingly increased after the treatment of the miR-27a-3p inhibitor (Figure 4g, h). Additionally, the complementary sequences of miR-27a-3p and ZNF268 were predicted through TargetScan (Figure 4i). The vectors containing wild-type and mutant sequences 3’-UTRs were transfected into cells and the results of dual-fluorescein reporter assay proved that ZNF268 was the direct target of miR-27a-3p (Figure 4j). This work also investigated the anti-tumor performances of miR-27a-3p using EdU assay (Figure 4i), and the results were consisted with the predication that miR-27a-3p functioned as the tumor promoter in ccRCC. Additionally, the upstream lncRNA of miR-27a-3p was predicted by two programs, LncBaes and StarBase. In total, 15 candidates were selected and visualized by Cytoscape (Figure 5a). However, only Lnc-AC093157.1 expression was significantly correlated to CCRCC clinicopathological features with tumor tissues possessing higher AC093157.1 expression level (all *P* < 0.001; Figure 5b). The high AC093157.1 expression was positively correlated with miR-27a-3p (*P* = 0.024; Figure 5c). Meanwhile, Kaplan-Meier analysis results showed that AC093157.1 expression was negatively correlated with overall and disease-specific survival (Figure 5d). Furthermore, the AUC was 0.742 in predicting ccRCC tissues analysis (Figure 5d). The co-relationship between AC093157.1 and miR-27a-3p was subsequently investigated by q-PCR after si-AC093157.1 treatments. The AC093157.1 expression was inhibited by the si-AC093157.1 treatment (Figure 5f), which was accompanied with the decreased miR-27a-3p expression (Figure 5g). The positively relationship between miR-27a-3p and AC093157.1 proved that the miR-27a-3p expression was regulated by AC093157.1. And the expressions of ZNF268 was detected after different pretreatments to evaluate the accuracy of our predication. The ZNF268 expression was significantly elevated by the treatments of miR-27a-3p inhibitor, si-AC093157.1 and the combination (Figure 5h, i). All the evidences from both in vitro and in silico analysis confirmed that AC093157.1/miR-27a-3p axis was considered as the upstream ncRNA which regulating ZNF268 expression and tumor progression.

## Discussion

Currently, the inhibition of tumor progress has been broadly investigated, contributing to the clinical therapy for cancer patients[18]. ccRCC, as one of the most common malignant tumors, has put an enormous burden on social development, which desiring comprehensive explorations of pathogenesis[19]. Increasing evidence indicates that ZNF268 might play an essential role in the development of various tumors [20–22]. However, the biological function of ZNF268 in ccRCC has not been explored. We report that ZNF268 is downregulated in ccRCC tumors; a higher expression level is positively related to a favorable clinical prognosis. Moreover, overexpression of ZNF268 inhibits the proliferation, migration, and invasion of ccRCC cells both *in vitro* and *in vivo*. The cell apoptosis was enhanced by the overexpression pf ZNF268, which was confirmed by the flow assay and WB results of Bcl-2/Bax. Meanwhile, elevated ZNF268 expression activated the T_CM_ and T helper cells infiltration, inhibited the infiltration of T_reg_ in ccRCC, which assisting in suppressing the tumor progress. Mechanistically, the AC093157.1/miR-27a-3p axis was proposed as the potential upstream ncRNA pathway of ZNF268 in ccRCC.

ZNF268, as the protein-coding gene, is involved in nucleic acid binding and DNA-binding transcription factor activity. It encodes two protein isoforms, ZNF268a and ZNF268b29. ZNF268a, consisting of a KRAB domain and 24 zinc fingers, functions as a transcriptional repressor[23]. By interacting with IκB kinase (IKK) α, ZNF268a promotes the production of pro-inflammatory cytokines upon viral infection by helping to maintain the association between subunits of the IKK complex subunits[7]. In contrast, ZNF268b2 does not contain the KRAB domain and has been reported to promote cervical carcinogenesis by interacting with IKK, facilitating IKKα/β phosphorylation, and activating the NF-κB signaling pathway[21]. Additionally, ZNF268b2 acts as a transcriptional repressor that inhibits erythroid differentiation and tumor cell proliferation [22,24,25]. Our study reveals that ZNF268 overexpression effectively inhibits cell proliferation, migration, and invasion *in vitro*, and the results from the xenograft tumor model further confirm the tumor-suppressing performance of ZNF268. The potential mechanisms of ZNF268 anti-tumor function originated from the enhanced cell apoptosis, which had been confirmed by various experiments. Meanwhile, Kaplan-Meier survival curves show that CCRCC patients with high ZNF268 expression have better overall and disease-specific survival. All clinical and experimental evidence indicates that ZNF268 could be considered a tumor suppressor for ccRCC patients.

Numerous studies have proved that immune infiltration affects the clinical efficacy of cancer therapy and prognosis of patients [26,27]. This work explores the relationship between ZNF268 expression and the types of immune cell infiltration in ccRCC. The results indicate that ZNF268 is positively related to T_CM_ and T helper cells infiltration, suggesting a tumor-suppressing effect. As the defense system, the immune responses contribute to the various anti-tumor performances in cancer patients[28]. The T helper cells function is widely investigated in numbers of studies for the excellent immune activation in the body [28–30]. The effector T_H_ cells originated from T helper cells under stress can generated various cytokines, including IL-2, IL-4, et al, and further activate innate immune responses against kinds of damages, which assisting the anti-cancer therapy[30]. Meanwhile, the memory T_H_ cells generated from T helper cells construct the basis for the adaptive immunity, which effective preventing the tumor recurrence[31]. And the T_CM_ cells can be activated by the tumor cells and generated CD8^+^ T cells, which functioning as the tumor cells killer in immune system[32]. And the T_reg_ cells infiltration can be inhibited by ZNF268 expression, which also enhancing the anti-cancer performances[33]. It is considered that T_reg_ cells enhanced the vulnerability of tumor to free oxygen species, increasing the tumor resistance to therapy[33]. Meanwhile, the NK bright cells infiltration is also related with advanced stages of cancer[34]. The negatively co-relationship between ZNF268 expression and NK bright cells infiltration can contribute to the anti-cancer function. However, the detailed immune activation of ZNF268 remains in-depth investigation both *in vitro* and *in vivo*, which would be explored in the next stage of our work.

Additionally, the function of ncRNAs in tumor carcinogenesis has been investigated worldwide, including miRNAs, lncRNAs and circRNAs, which play a role in regulating gene expression[19]. We first explored the potential upstream miRNA in ZNF268 regulation by introducing four predicting programs, including miRWalk, microRNA, TargetScan, and StarBase. MiR-27a-3p was selected in our work due to its negative correlation with ZNF268 expression. Various reports confirm that miR-27a-3p promotes proliferation and metastasis of renal cell carcinoma [35,36], consistently with our findings. Moreover, this work also provides supporting evidence on the ZNF268-regulating function of miR-27a-3p. The related lncRNA is analyzed in this study using the two databases of StarBase and LncBase. LncRNA-AC093157.1 functions as the predicted upstream lncRNA of miR-27a-3p/ZNF268 has been considered an oncogene in ccRCC for the negative association with tumor prognosis, which is also proved by results of experiments *in vitro*. Unlike the ceRNA hypothesis, the positive relationship between AC093157.1 and miR-27a-3p will be attributed to stabilizing the microprocessor complex subunit DGCR8 [37,38]. Finally, AC093157.1/miR-27a-3p/ZNF268 axis is determined as the potential regulatory pathway in ccRCC.

## Conclusion

**Figure 6.**
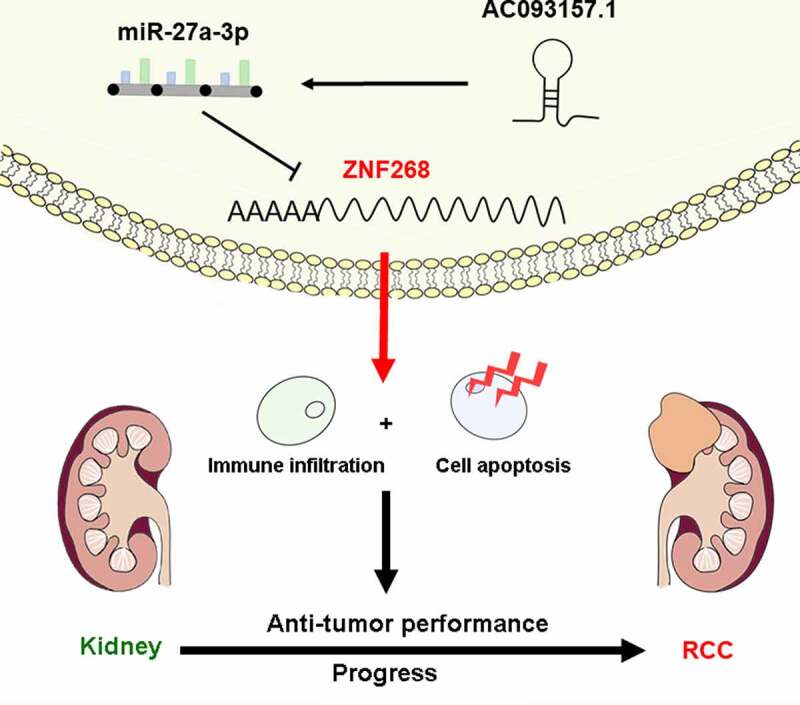
Schematic representation for the regulatory AC093157.1/miR-27a-3p/ZNF268 axis in carcinogenesis of ccRCC.

In conclusion, our findings elucidate that ZNF268 expression is significantly downregulated in ccRCC and related to the poor prognosis of patients. Mechanistically, the AC093157.1/miR-27a-3p axis is identified as the upstream ncRNAs in regulating ZNF268 expression, which would activate immune infiltration (Figure 6). Collectively, this work proves that ZNF268 functions as a considerable inhibitor of tumor prognosis and has promising prospects in ccRCC therapy.

## Supplementary Material

Supplemental Material

## Data Availability

Raw data were generated at the Central Laboratory of Shanghai Tenth’ Peoples’ Hospital. Derived data supporting the findings of this study are available from the corresponding author on request.
