# Supplementary material for: Noncoding-RNA mediated high expression of zinc finger protein 268 suppresses clear cell renal cell carcinoma progression by promoting apoptosis and regulating immune cell infiltration
Source: Bioengineered. 2022 Apr 23;13(4):10467–81. doi: 10.1080/21655979.2022.2060787 (PMC9161828; doi:10.1080/21655979.2022.2060787)
Supplement: Supplemental Material [file KBIE_A_2060787_SM6657.zip › Table S3clean.docx]

| miR-RNAs | *r*-value | *P*-value |
| --- | --- | --- |
| hsa-miR-675-3p | 0.148 | <0.001 |
| hsa-miR-641 | 0.04 | 0.365 |
| hsa-miR-33a-5p | **-0.117** | **0.008** |
| hsa-miR-330-5p | -0.058 | 0.188 |
| hsa-miR-486-5p | -0.013 | 0.764 |
| hsa-miR-3164 | -0.035 | 0.424 |
| hsa-miR-134-5p | 0.035 | 0.431 |
| hsa-miR-181b-5p | 0.09 | 0.042 |
| hsa-miR-212-3p | 0.004 | 0.929 |
| hsa-miR-3167 | -0.071 | 0.107 |
| hsa-miR-7-5p | -0.051 | 0.253 |
| hsa-miR-30d-5p | -0.032 | 0.472 |
| hsa-miR-3171 | 0.066 | 0.137 |
| hsa-miR-584-5p | 0.186 | <0.001 |
| hsa-miR-1294 | 0.078 | 0.077 |
| hsa-miR-128-3p | 0.005 | 0.903 |
| hsa-miR-556-5p | -0.009 | 0.641 |
| hsa-miR-181c-5p | 0.235 | <0.001 |
| hsa-miR-132-3p | 0.034 | 0.438 |
| hsa-miR-27a-3p | **-0.138** | **0.002** |
| hsa-miR-33b-5p | **-0.105** | **0.017** |
| hsa-miR-513a-5p | -0.016 | 0.713 |
| hsa-miR-3118 | 0 | 0 |
| hsa-miR-216a-3p | 0.022 | 0.627 |
| hsa-miR-3140-3p | -0.045 | 0.313 |
| hsa-miR-181d-5p | 0.204 | <0.001 |
| hsa-miR-129-5p | 0.05 | 0.257 |
| hsa-miR-299-5p | 0.043 | 0.327 |
| hsa-miR-5581-3p | 0.042 | 0.346 |
| hsa-miR-337-3p | 0.072 | 0.105 |
| hsa-miR-942-5p | 0.062 | 0.16 |
| hsa-miR-181a-5p | 0.128 | 0.004 |
| hsa-miR-27b-3p | 0.029 | 0.515 |

**Table S3** The results of Spearman analysis between the expressions of included miR-RNAs and ZNF268 expression.
