# Supplementary figures and images for: Noncoding-RNA mediated high expression of zinc finger protein 268 suppresses clear cell renal cell carcinoma progression by promoting apoptosis and regulating immune cell infiltration
Source: Bioengineered. 2022 Apr 23;13(4):10467–81. doi: 10.1080/21655979.2022.2060787 (PMC9161828; doi:10.1080/21655979.2022.2060787)

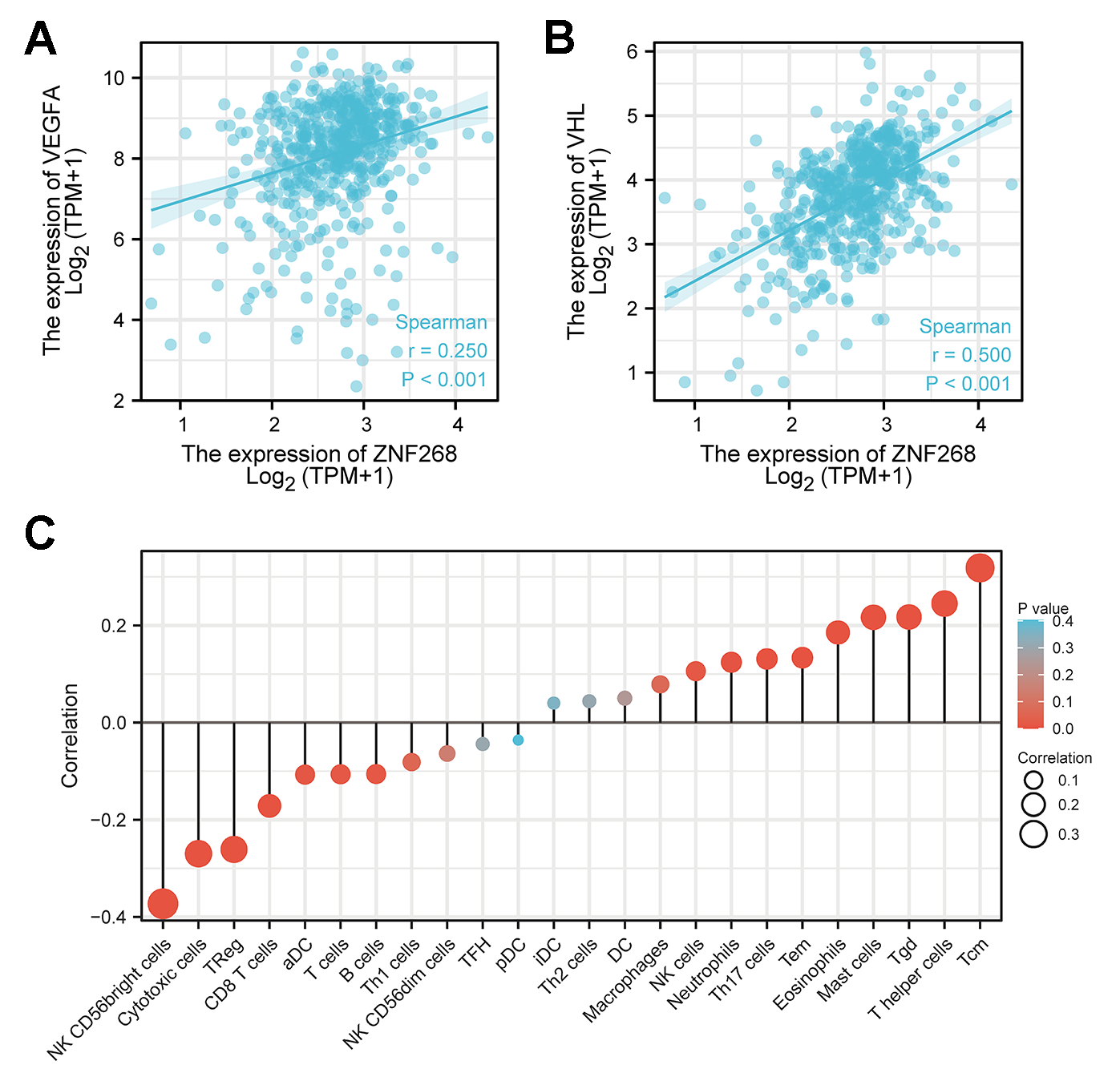

Supplement: Supplemental Material [file KBIE_A_2060787_SM6657.zip › Figure S1.tif]

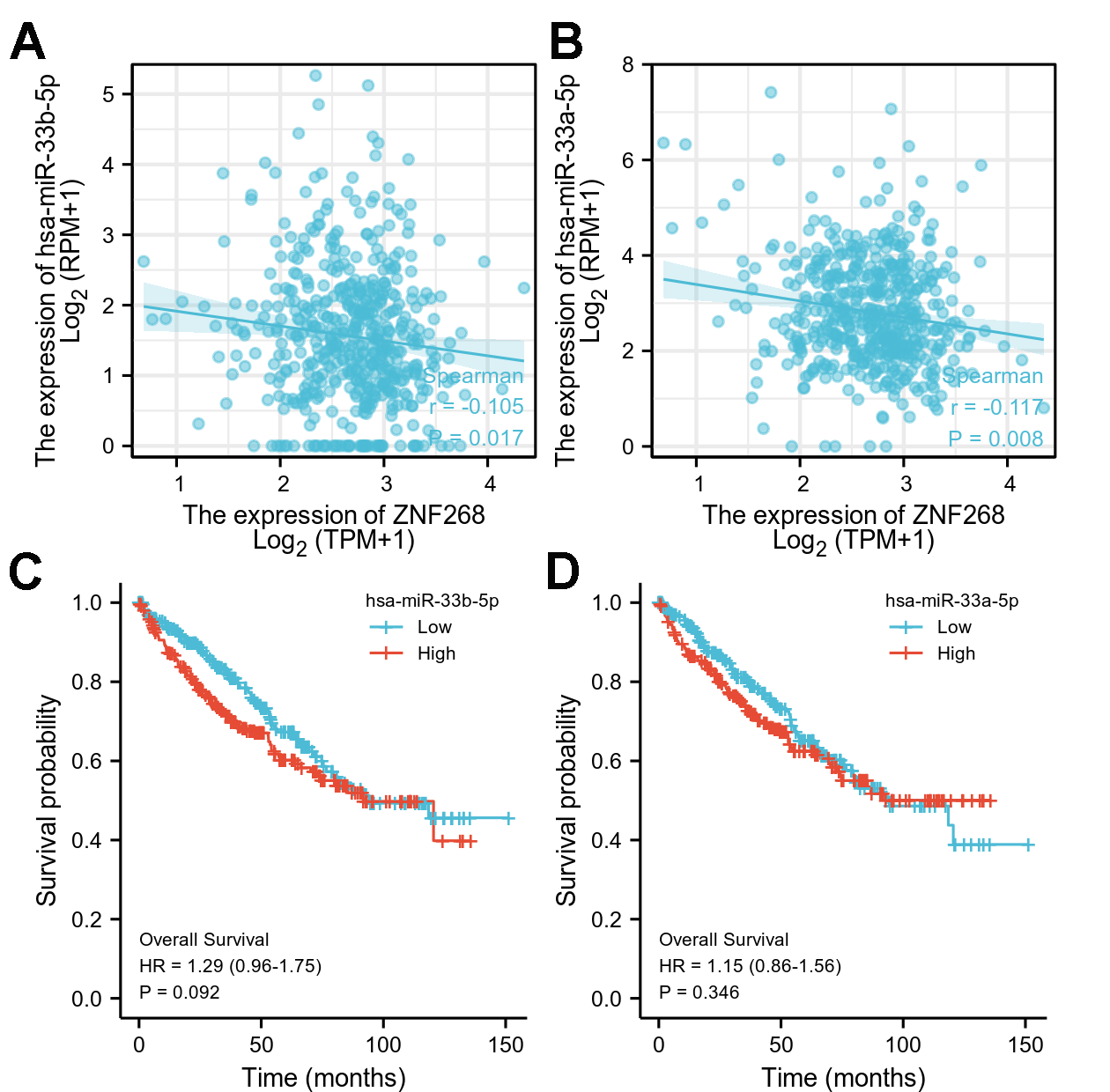

Supplement: Supplemental Material [file KBIE_A_2060787_SM6657.zip › Figure S2.tif]

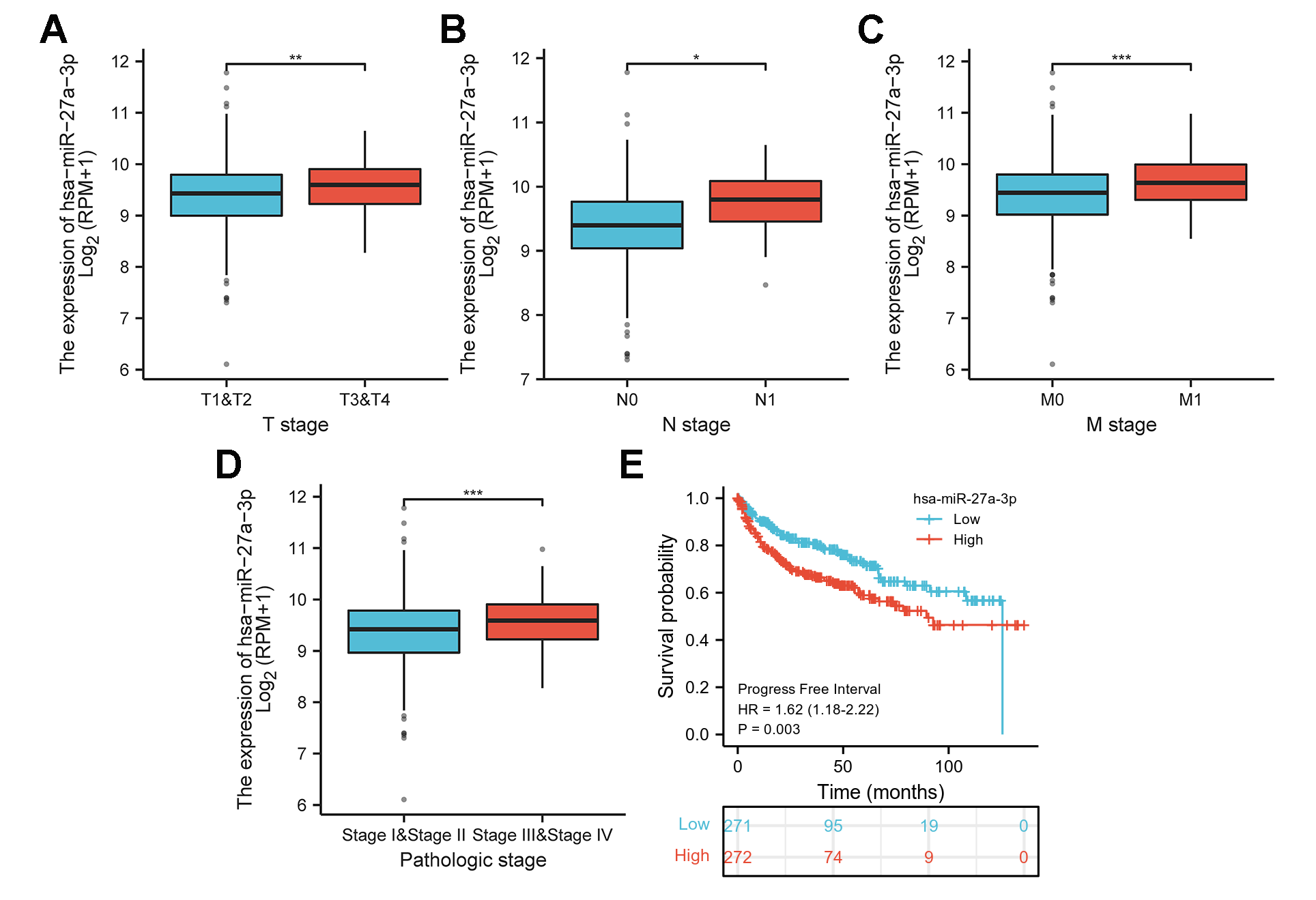

Supplement: Supplemental Material [file KBIE_A_2060787_SM6657.zip › Figure S3.tif]
